# Supplementary material for: Prevalence and determinants of mental health problems experienced by school-going adolescents in Sri Lanka
Source: Glob Ment Health (Camb). 2025 Sep 17;12:e104. doi: 10.1017/gmh.2025.10055 (PMC12509160; doi:10.1017/gmh.2025.10055)
Supplement: Mudunna et al. supplementary material [file S2054425125100551sup001.zip › Supplement_1.docx]

**Appendix 1**

*Sample Size Calculation*

The sample size was calculated using the World Health Organization Sample size determination in health studies practical manual (Lwanga and Lemeshow 1991) to estimate a population proportion (in this study, clinically significant symptoms of depression and anxiety). Data used in these calculations were retrieved from results identified in previous relevant research. Since there was no widely used previous research of the same type in this setting, relevant results from previous studies were used as a guide only; where there was no relevant information, assumptions were made. P=0.5 was used to ensure the sample size appropriate for any population portion. A confidence level of 95% with an absolute precision required on either side of the proportion to be 5% was used. With these factors, the number arrived at per the WHO sample size calculator is 384.

A conservative response rate of 90% was assumed to adjust for sample size, as past studies in this population indicated high response rates of around 98% (Agampodi et al. 2011; Perera et al. 2006; Rodrigo et al. 2010). A further 10% was added to account for missing data and then multiplied by 2 to count for “design effect” because a multiple-stage sampling method was used. The final calculated minimum sample size was 844.

*Procedure*

A rigorous, systematic method of data collection was followed.

First, approval to conduct fieldwork for this study was obtained from the Western Province Department of Education and the Line Ministry of Education in Sri Lanka. Next, permission was obtained from the relevant Zonal Education Office in the Gampaha Zone of Gampaha District, Sri Lanka. Third, the research team obtained permission to conduct the study in each of the seven schools from the respective principals.

Once permissions were obtained to conduct the study, consent was sought from parents of participants under the age of 18. Parents were sent an information sheet and consent form for signed consent for their child to participate in the survey. Adolescents who returned parental signed consent forms were eligible for participation. All adolescents, whether over the age of 18 or under the age of 18 with consent from parents, were provided an information sheet prior to survey completion. Next, those completing paper-based surveys were provided a paper-based assent form for signed assent and those completing online surveys were required to provide online assent prior to survey completion. All students in the selected schools were notified that participation in the study is entirely voluntary and that the survey is not part of their standard syllabus. The ability to withdraw consent and not complete the survey at any point was clearly explained to all participants.

**Appendix II**

*Conceptual Framework*

*
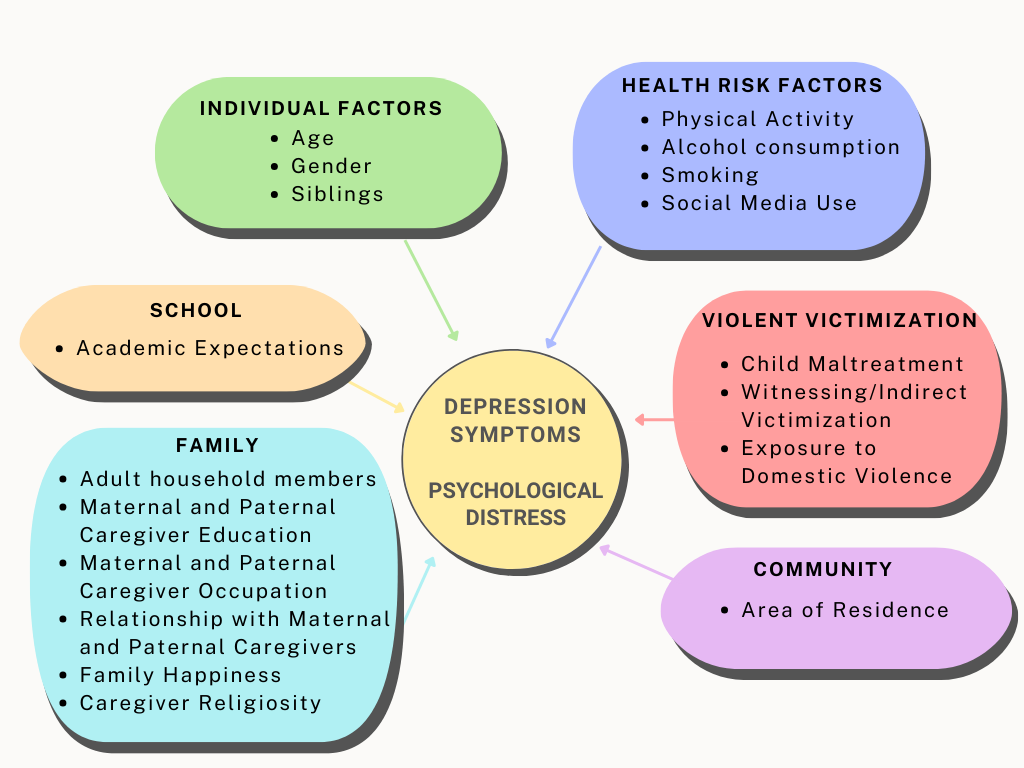
*

**References**

**Agampodi TC, Agampodi SB and Fonseka P** (2011) Prevalence of Mental Health Problems in Adolescent Schoolchildren in Galle District, Sri Lanka: Eight Months After Tsunami. *Asia-Pacific Journal of Public Health* **23**(4)**,** 588-600.

**Lwanga SK and Lemeshow S** (1991) *Sample Size Determination in Health Studies*. Geneva: World Health Organization.

**Perera B, Torabi M, Jayawardana G and Pallethanna N** (2006) Depressive Symptoms among Adolescents in Sri Lanka: Prevalence and Behavioral Correlates. *Journal of Adolescent Health* **39,** 144-146. <https://doi.org/https://doi.org/10.1016/j.jadohealth.2005.10.013>.

**Rodrigo C, Welgama S, Gurusinghe J, Wijeratne T, Jayananda G and Rajapakse S** (2010) Symptoms of anxiety and depression in adolescent students; a perspective from Sri Lanka. *Child and Adolescent Psychiatry and Mental Health* **4**(10). <https://doi.org/10.1186/1753-2000-4-10>.
