# Supplementary figures and images for: Prevalence and determinants of mental health problems experienced by school-going adolescents in Sri Lanka
Source: Glob Ment Health (Camb). 2025 Sep 17;12:e104. doi: 10.1017/gmh.2025.10055 (PMC12509160; doi:10.1017/gmh.2025.10055)

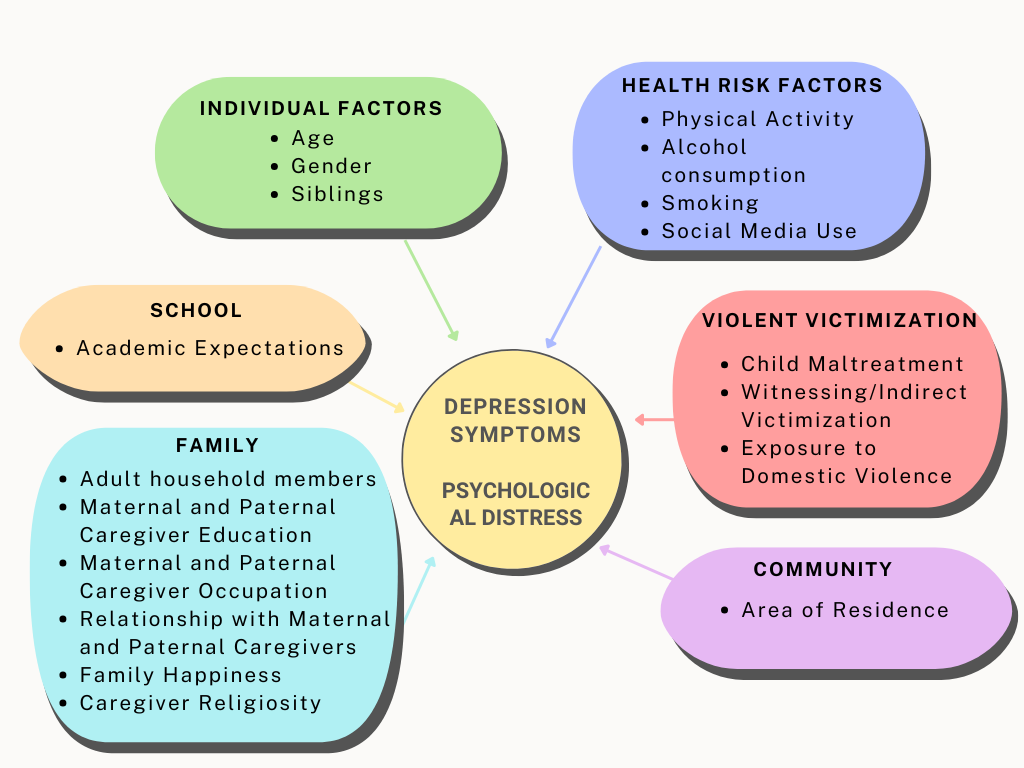

Supplement: Mudunna et al. supplementary material [file S2054425125100551sup001.zip › Supplementary_File_S1.png]
